# Variation in urine output from shelter cats is explained by shelter location, not kennel size

**DOI:** 10.1371/journal.pone.0320130

**Published:** 2025-04-08

**Authors:** Allison Andrukonis, Nathaniel J. Hall

**Affiliations:** 1 School of Animal Sciences, Virginia Tech, Blacksburg, Virginia, United States of America; 2 Department of Animal and Food Sciences, Texas Tech University, Lubbock, Texas, United States of America; Benha University, EGYPT

## Abstract

Monitoring and decreasing stress in cats housed in animal shelters is essential for maintaining adequate welfare. Urine output is a potential indicator of shelter cat stress. The present study aimed to evaluate the impact of the provision of extra space using a portal connecting two kennels and daily petting on urine output at two different municipal shelters in the United States. Cats (n =  59) were randomly assigned to one of four groups: Control, Portal, Petting, and Portal and Petting. Litter clumps were weighed daily for five days as a measure of urine output. Additionally, cats were given a daily Cat Stress Score. Experimental group did not significantly predict urine output nor Cat Stress Score. However, living at Shelter B significantly predicted increased urine output. The results from the present study suggest that shelter-related factors might impact urine output of cats more than kennel size or petting alone.

## Introduction

United States (US) animal shelters house approximately 3.2 million cats (*Felis silvestris catus*) annually [1]. The majority of those cats are either surrendered by their caregiver or found as strays [2]. While in the shelter, many cats are singly housed in kennels [3,4]. The housing guidelines outlined by The Association of Shelter Veterinarians (ASV) state that adequate cat housing must allow for postural adjustments as well as enough space for the separation of areas for elimination from feeding and resting areas [5]. The ASV also recommends housing with multiple compartments to facilitate cleaning and to give the animal choice in their environment. Although multi-compartment kennels have been suggested to allow for a separate area for elimination, the impact of multiple compartments and additional space on elimination has yet to be evaluated. Many shelters have single-compartment kennels, and it is not financially feasible to purchase new kennels. However, there are commercially available cat portals as well as do-it-yourself PVC portals that can be used to connect two single-compartment kennels [6,7]. Previous research found that retrofitting single-compartment kennels to be two compartments decreased length of stay and increased adoption rates in cats [8]. Additionally, multi-section cages have been correlated with decreased risk of upper respiratory infection in shelter cats [9].

Upper respiratory tract infections (URIs) have been the primary focus of illness research in shelter cats [10–15]. However, recently, there has been increased interest in the urinary health of both owned and shelter-housed cats [16–18]. Urinary problems are one of the leading reasons for owners to seek veterinary care for their cats [19,20]. Additionally, periuria, or inappropriate urination, is also a leading reason for relinquishment of cats to animal shelters [21]. Urinary problems are frequently caused by feline idiopathic cystitis (FIC) [22]. FIC is still poorly understood, but is thought to be the result of the complex interaction between the bladder, nervous system, adrenal glands, and the physical and social environment in which the cat lives [22,23]. Although FIC can resolve on its own, recent estimates suggest it reoccurs in 36-58% of cats [24,25]. Recurrent FIC is difficult to treat and can have detrimental impacts on cat welfare [22]. A recent survey of small animal veterinarians in the US found that 53% had been asked to perform a euthanasia for FIC and 10% had recommended euthanasia for a cat with FIC [26]. There are several risk factors associated with the development of FIC, including being male [27–29], exposure to social and environmental stressors [27–32], being overweight [27,29–32], consuming dry food [28], and indoor confinement [30–33]. Additionally, according to owner reports, cats with FIC display more fearful and nervous behaviors compared to healthy cats and cats with other diseases [30,31,34]. Shelter cats are exposed to both social and environmental stressors [4,35–37], confined to small, indoor spaces [10,37,38], and are often fed dry food [37–39], which might make them more prone to urinary problems.

Monitoring stress is an important part of improving quality of life for shelter cats. However, there is no comprehensive method for evaluating shelter cat stress [40]. Animal shelters are frequently underfunded and understaffed. As such, methods of monitoring stress as well as approaches to alleviating stress should be time and cost effective. The Cat Stress Score (CSS), a measure reliant on cat behavior and posture, has been used as an indicator of stress in shelter cats [4,17,41–43]. However, some researchers have suggested that the CSS, which scores cats from 1 (fully relaxed) to 7 (terrified) is actually a measure of fear, not stress [44]. Additionally, given the number of staff responsible for daily husbandry, shelters could benefit from an easily collected, objective measure of stress such as urine output.

In order to decrease the stress associated with unfamiliar handling during daily husbandry, the ASV recommends spot cleaning cat kennels [5]. Spot cleaning allows the cat to remain in their kennel while the staff removes soiled bedding, urine and feces, and food and water are replaced. Litter box cleaning is a necessary part of daily husbandry and the contents of the litter may be an indicator of stress in cats [5,45]. Urine output has been shown to be suppressed one day after being moved to a new kennel, suggesting it could be an indicator of stress [16,17,45]. A recent study found that urine output can be reliably measured via clump weight collected during daily husbandry in shelter cats [16]. Additionally, another study found that urine suppression was significantly decreased in cats that received daily gabapentin, a medication used to decrease stress in cats [46,47], providing additional evidence that urine output might be an indicator of stress in shelter cats [17]. Not only has medication been used to decrease stress in shelter cats, but human interaction has also been utilized [48].

Human interactions in the form of gentle petting, also called gentling, have been shown to improve behavior and immunity in shelter cats [12,13,15]. Shelter cats who received petting were more likely to be rated as content, as compared to anxious or frustrated, had increased secretory immunoglobulin A (S-IgA) [13], and were less likely to develop an URI [12]. Through a series of experiments examining best practices in petting shelter cats, Liu and colleagues [49] found that one, six-minute petting session daily predicted greater time spent in the front of the kennel, compared to three, two-minute sessions daily. Interestingly, if vocalizations were provided during the petting, cats were more likely to spend time in their litter area, which may be a sign of stress [50]. Additionally, cats who received petting sessions of six or nine minutes were more likely to purr, eat, and drink [49]. The impact of petting on urine output, however, has yet to be evaluated.

The aim of the present study is to expand the current research on urine output as a potential indicator of stress in shelter cats by providing stress-reducing interventions. More specifically, the study aims to: examine the impact of the provision of extra space through portals and petting on urine output and cat stress score. Additionally, the study aims to examine the relationship between shelter location and intake type (owner surrender vs. stray) on urine output and Cat Stress Score.

## Materials and methods

### Ethical approval

All study procedures were reviewed and approved by the Texas Tech Institutional Animal Care and Use Committee (T19062-06) and the Virginia Tech Institutional Animal Care and Use Committee (22-166).

### Animals and housing

Cats of unknown breed (n =  59; approximately 6 months to 10 years of age; 53% female), who appeared healthy were enrolled. Cats were housed at two different open-admission, municipal shelters. Shelter A, located in the South-West United States, had an annual intake of 1,930 cats in 2022. Data collection for Shelter A occurred from December 2021 to March 2022. Cats (n =  18) in Shelter A were singly housed in 71.12 cm x 59.69 cm x 67.31 cm steel and plexiglass kennels with an attached litter area (26.67 cm x 59.69 cm x 67.31 cm). The cat cages had a 55.88 cm x 27.94 cm shelf, 41.91 cm off the ground. The cat kennels also included a hide box. Occasionally a small plastic ball with a bell inside or a ping pong ball would be in the cages. The cat kennels were up against windows, facing the parking lot and a highway. The litter areas did not backup to windows. Members of the community were allowed to view the cats at Shelter A Monday-Saturday, from 10am – 7pm.

Shelter B, located in the Mid-Atlantic United States, had an annual intake of 1,500 cats in 2022. Data collection for Shelter B occurred from September 2022 to April 2023. Cats in Shelter B (n =  41) were singly housed in 57.79 cm x 70.49 cm x 71.12 cm stainless steel kennels. Each kennel had a towel covering the bottom of the kennel. The cat kennels also included a hide box. Members of the community did not have access to the cats enrolled in the study at Shelter B. Individual cats were enrolled in the study for six days. The research team, rather than shelter staff, cleaned the cages and fed and watered the cats enrolled in the study at both locations to ensure experimental integrity. Shelter staff at both locations categorized their respective cats by their intake type (stray [n =  30], owner surrender [n =  28], seized [n =  1]).

### Experimental groups

Cats were randomly assigned to one of four groups: Petting (n =  14), Portal (n =  15), Petting and Portal (n =  15), and Control (n =  15). Based on the best practices outlined by Liu and colleagues [49], cats in the Petting groups received eight minutes of quiet, consent-based petting on days 0-4. During the petting sessions, the researcher placed their hand in the kennel and started the timer. If the cat made contact with the researcher’s hand, the researcher would pet the cat. If the cat moved away from the researcher’s hand, the petting stopped. Regardless of the cat making contact with the researcher’s hand, the researcher kept their hand in the kennel for the full eight minutes. The researcher preforming the petting was kept consistent throughout the study. Cats in the Portal groups were housed for the duration of the study in a retrofitted kennel. The original single kennels were connected using a Shor-Line Kat Portal and the protocols outlined by Wagner and colleagues [51–53], effectively doubling their kennel space. Cats in the Petting and Portal groups received daily petting and were housed in retrofitted double kennels. Cats in the Control group were housed in the original single kennels and did not receive any petting.

### Data collection

On day 0 of the study, cats were transferred from their holding area to the study location. The majority of cats (n =  57) moved one day after arriving at the shelter. Two cats were moved later, on days two and three respectively. Cats were weighed before they were placed in their new kennel. For the following five days, food and water consumption and urine clump weight were measured daily. Cats remained in the same kennel for the duration of the study.

#### Urinary measurement.

Cats were given a clean litter box daily. Litterbox and litter type were kept consistent across locations. The average total litter and litter box weight was 1197.6g (range: 1010-1530g). Throughout the study, litter clump weight was measured daily between the hours of 7:30 and 9:30 am local time, using the protocol outlined in Andrukonis and colleagues [16]. Clumps were collected and weighed using a slotted litter scoop and paper weigh boats.

#### Feces.

The presence or absence of feces was recorded

#### Food and water consumption.

Cats were given pre-weighed fresh dry food and water daily. The remaining food and water leftover from the day before were weighed to calculate the amount consumed. If the cat spilled their water, then the daily water consumption was left blank.

#### Cat Stress Score and Spectrum of Fear, Anxiety, and Stress.

Prior to daily husbandry, a brief (6-20 second) video was recorded of each cat. The cats did not have prior exposure to the research team before recording the first video. The videos were then used to assign each cat a Cat Stress Score (CSS) [41] and Spectrum of Fear, Anxiety, and Stress (FAS Spectrum) score [54]. CSS is an observational stress classification based on behavioral and postural attributes [41]. Possible scores on each of the 11 attributes range from 1 (fully relaxed) to 7 (terrified). Similar to the scoring methods used by Dydball and colleagues [38], each attribute was scored individually. An overall CSS was obtained through averaging all 11 attributes for a cat on a single day. Similar to the CSS, the FAS Spectrum is an observational stress classification. To the authors’ knowledge the FAS Spectrum has not been previously used in its full form in an empirical study. However, a slightly modified version of the FAS Spectrum had excellent interrater reliability (ICC = .94) when tested using video clips of shelter cats [55]. The FAS Spectrum provides one overall score. Each of the possible scores is associated with a behavioral and postural description and 1-3 pictures. The descriptions include postures previously found to be associated with both positive (e.g., tail up [56]) and negative (e.g., ears back [57]) affect. The possible scores on the FAS Spectrum are 0 (Green; Relaxed), 0-1 (Green; Perked/ Interested/ Anxious?), 1 (Green; Mild/ Subtle Signs), 2-3 (Yellow; Moderate Signs), 4 (Red; Severe Signs – Flight/ Freeze/ Fret), and 5 (Red; Severe signs—fight/aggression). Score of 0-1 and 2-3 were coded as 0.5 and 2.5, respectively.

A randomly selected portion of the videos (20%) were double coded. Coder agreement was calculated using intraclass correlation coefficient (ICC). The ICC for the FAS Spectrum and CSS was 0.805 and 0.909, respectively.

### Data analysis

All data analyses was done in R [58]. Prior to data analysis, one cat was removed from analysis because she had a unique intake type (i.e., seized). Additionally, incomplete data points were removed. A Spearman’s rank correlation was used to determine the relationship between Cat Stress Score (CSS) and the Spectrum of Fear, Anxiety, and Stress (FAS Spectrum) score. A linear mixed-effects model was run on urine output with fixed effects of CSS, days in the study, experimental group (Petting, Portal, Petting and Portal, and Control), water intake, food intake, presence of feces, weight, sex, intake type (stray, owner surrender), and shelter location, and a random effect of participant. The model was reduced through sequential use of the drop function using the F test [58]. The drop function compares all possible single-term deletions via nested model comparison. The full model was tested for multicollinearity using the variance inflation factor (VIF) [59]. All predictor variables had an adjusted GIF (GVIF^(1/(2 * Df))) of less than 2, suggesting collinearity was not a concern [60]. Additionally, a linear mixed-effects model was run on CSS with fixed effects of days in the study, experimental group, water intake, food intake, presence of feces, weight, sex, intake type, and shelter location, and a random effect of participant. The full model was also tested for multicollinearity using VIF and reduced used the drop function. All predictor variables had an adjusted GVIF (GVIF^(1/(2 * Df))) of less than 2, suggesting collinearity was not a concern. Finally, two Wilcoxon Rank Sum Tests were used to compare urine output and CSS in cats in the petting groups who made contact with the researcher’s hand (and thus received petting) as compared to those that did not.

## Results

The average daily amount of food and water consumed was 49.6g (range: 0-99g) and 128.9g (range: 4-339g), respectively. The average litter clump weight across all conditions and both shelter locations was 179.35g (range: 0-504g). Table 1 shows the median clump weight by days in the study and shelter location. The average daily CSS in the current study was 3.08 (range: 1.50-5.45). A CSS of 3 indicates a cat is weakly tense [41]. The average daily FAS Spectrum score in the current study was 1.14 (range: 0-4), indicating the cats were displaying mild/subtle signs of stress [54].

**Table 1:** Median clump weight at Shelters A and B across days in the study.

Clump Weight (g) Median (IQR)					
	Day 1	Day 2	Day 3	Day 4	Day 5
Shelter A	110 (43-145)	127 (103.5-160.25)	115.5 (62.25-131.25)	156 (91-165)	109 (77.5 - 156.5)
Shelter B	224 (145-282)	185 (152-246.5)	187 (140-227)	169.5 (144.25-207)	188 (147-233)

**Fig 1:**
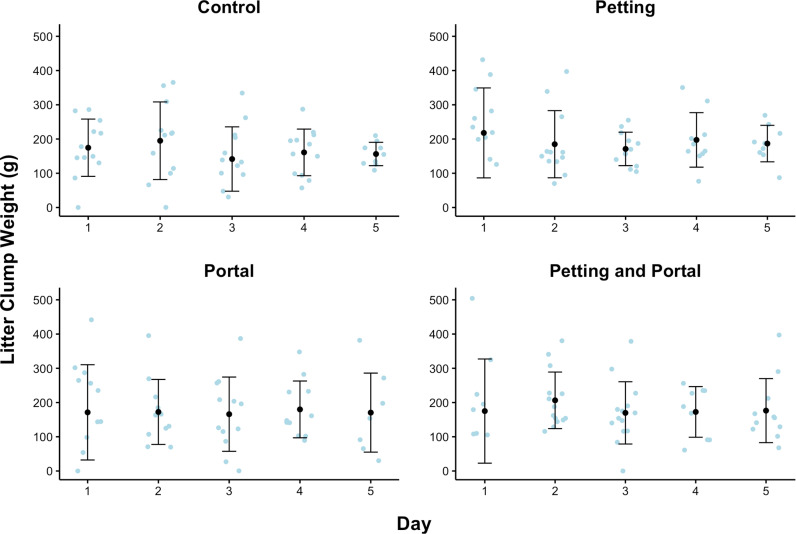
Litter clump weight across experimental groups and days in the study. For the final analysis, the Petting and Portal group had 15 participants, and the Control, Petting, and Portal groups had 14 participants each. The black dots represent the mean. The bars represent one standard deviation above and below the mean. The blue dots represent individual data points. Experimental condition did not significantly predict litter clump weight.

**Fig 2:**
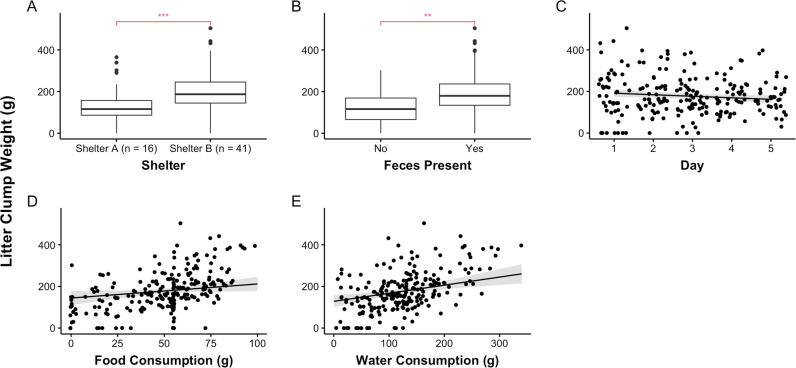
The variables that significantly predicted litter clump weight in shelter cats. A) Shelter B had significantly heavier litter clumps (p <  0.001) than Shelter A. The horizontal lines represent the median. The box represents the interquartile range. The dots represent outliers. B) Cats who defecated had significantly heavier litter clumps (p =  0.003) than cats who did not. C) Fewer days in the study predicted greater urine output (p = .034). The black line represents the line of best fit based on the reduced model with marginal means. The gray shaded area shows the prediction interval that accounts for the uncertainty in the random effects. D) Greater food consumption was significantly related to increased litter clump weight (p =  0.039). E) Greater water consumption was significantly related to increased litter clump weight (p <  0.001).

Cat Stress Scores (CSS) were significantly related to scores on The Spectrum of Fear, Anxiety, and Stress (*r* =  0.849, *p* <  0.001). Contrary to the hypothesis, experimental group (i.e., Control, Petting, Portal, or Petting and Portal) did not significantly predict litter clump weight. Fig 1 shows litter clump weight across experimental groups and days in the study. Additionally, litter clump weight was not predicted by CSS, intake type, sex, nor cat weight. Greater litter clump weight was significantly predicted by greater food (*F* (1, 208.788) =  4.322, *p* =  0.039) and water consumption (*F* (1, 220.872) =  13.478, *p* <  0.001), defecating (*F* (1, 220.671) =  8.833, *p* =  0.003), fewer days in the study (*F*(1,183.502) =  4.556, *p* = .034), and being housed at Shelter B (*F* (1, 46.017) =  14.692, *p* <  0.001). Fig 2 shows the variables that significantly predicted litter clump weight in the shelter cats.

**Fig 3:**
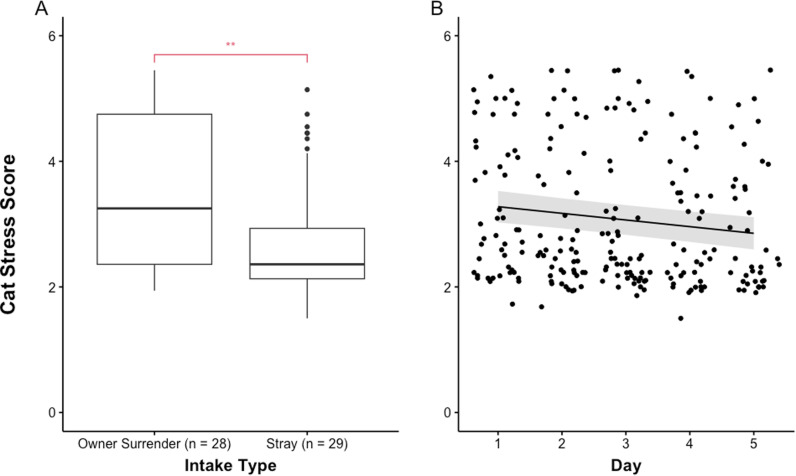
Cat Stress Score (CSS) was significantly predicted by intake type and days in the study. Possible scores range from 1 (fully relaxed) to 7 (terrified). A) Cats who entered the shelter as a stray had significantly lower CSS (*p* =  0.002) than cats who were surrendered to the shelter by their caregiver. The horizontal lines represent the median. The box represents the interquartile range. The dots represent outliers. B) More days in the study predicted lower CSS (*p* <  0.001). The black line represents the line of best fit based on the reduced model with marginal means. The gray shaded area shows the prediction interval that accounts for the uncertainty in the random effects.

CSS was not significantly predicted by experimental group, food and water consumption, the presence of feces, cat weight, sex, nor shelter location. However, a lower CSS was significantly related to more days since entering the study (*F* (1, 180.129 =  22.874.417, *p* <  0.001) and coming into the shelter as a stray (*F* (1, 55.373) =  11.082, *p* =  0.002). See Fig 3 for a visual representation of the variables that significantly predicted CSS.

**Fig 4:**
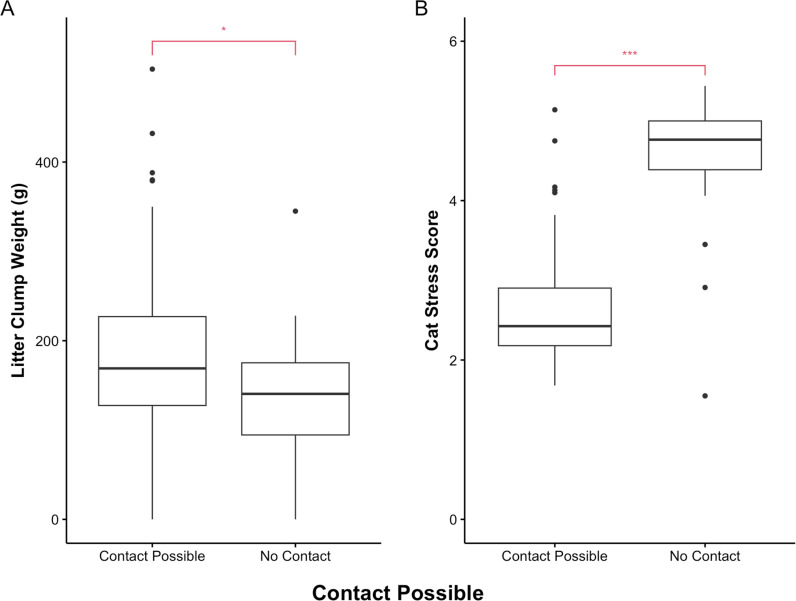
Comparison of cats in the petting groups who contacted the researcher’s hand vs. those who did not. Cats in the petting groups who made contact with the researcher’s hand during daily petting had significantly (A) heavier litter clump weights (*p* =  0.040) and (B) lower cat stress scores (*p* < .001). The horizontal lines represent the median. The boxes represent the upper and lower quartile. The dots represent outliers.

Although experimental group did not predict litter clump weight, when looking specifically at cats who received petting, cats who made contact with the researcher’s hand, thus receiving petting, had significantly heavier litter clump weights (*W* =  702.5, *p* =  0.040) and significantly lower cat stress scores (*W* =  105.5, *p* <  0.001) than cats who did not contact the researcher’s hand. Fig 4 shows a visual representation of the relationship between making contact with the researcher’s hand and urine output and CSS.

## Discussion

Experimental group (i.e., Control, Petting, Portal, or Petting and Portal) did not predict litter clump weight nor Cat Stress Score (CSS). However, greater litter clump weight was significantly predicted by greater food and water consumption, defecating, fewer days in the study, and being housed at Shelter B. Additionally, lower CSS was significantly predicted by more days since entering the study and coming into the shelter as a stray.

The lack of significant difference in urine output from cats housed in kennels with a portal vs. those without supports previous research on the impact of kennel size by Stella and colleagues [45,61]. In a series of two studies, the research team evaluated the impact of an unmanaged vs. managed macro environment and an unenriched vs. enriched micro environment in traditionally sized kennels [45] and larger kennels [61]. In both studies, the research team recorded sickness behaviors and in-kennel behavior. Cats in the larger kennel study behaved similarly to cats in the traditionally sized kennels, indicating no impact of kennel size on sickness and in-kennel behaviors.

However, both studies found that cats living in a controlled macro environment (i.e., consistent and predictable husbandry and limited noise disturbances from people, dogs, etc.) acclimated more quickly, as evidenced by a decline in sickness behaviors and time in the hide box and an increase in affiliative behaviors [45,61]. This supports the finding in the present study that shelter location predicted urine output. Cats from Shelter A were housed in kennels with windows that faced the parking lot of the shelter. Members of the public were also able to visit the cats from 10am – 7pm Monday- Saturday. Additionally, the cat room was attached to the shelter lobby, so cats could frequently hear and see humans as well as dogs. Although husbandry was kept consistent, there were likely numerous disturbances throughout the day. In contrast, cats in Shelter B were housed in a room with no windows. Their section of the shelter was not available to the public, and dogs could not be seen and rarely heard, limiting the number of disturbances. Although sound was not recorded in the present study, it is likely that noise levels were higher in Shelter A. Increased noise has been correlated with increased stress behavior in shelter cats [36]. Thus, it is probable that the lack of control in the macro environment and noise level of Shelter A may have contributed to the decreased litter clump weight. It is worth noting that there were only 16 cats from Shelter A included in the final analysis. The small sample size makes it difficult to draw meaningful conclusions on the impact of kennel size specifically for Shelter A. However, a previous study on urine output in shelter cats also conducted at Shelter A found similar urine clump weights [16]. On Day 1, cats at Shelter A in the present study cats had a median clump weight of 110g (IQR = 43-145g). Similarly, on Day 1 in the previous study, cats had a median clump weight of 114g (IQR = 70-127g).

The results from the present study suggest that shelter cats might benefit from having a controlled macro environment [45,61]. However, having a controlled macro environment might require shelters to prevent or greatly limit the amount of people who can pass through the cat rooms. A survey of recent cat adopters found that 94% mentioned that having the ability to directly interact with their cat was important to them when selecting the cat they wanted to adopt [62]. An earlier survey of cat adopters found that 74% felt the ability to enter the cat’s kennel was important when selecting a cat [4]. Thus, limiting public access to cats might delay or prevent their adoption. Future research should explore the impact of limiting disturbances on stress, length of stay, and adoption outcomes in shelter cats.

Contrary to our hypothesis, increased litter clump weight was predicted by fewer days in the shelter. Interestingly, the increased urine output on Day 1 was only seen in Shelter B. In fact, the median clump weight on Day 1 for cats in Shelter B was two times heavier than the median clump weight on Day 1 for cats in Shelter A. The median clump weight on Day 1 for cats in Shelter A was 110g, and the median clump weight for the subsequent days was 116.5g. Whereas, the median clump weight on Day 1 for cats in Shelter B was 224g, and the median clump weight for subsequent days was 180g. One potential explanation for the higher urine output on Day 1 in Shelter B is that the cats did not like the type of litter Shelter B used. Cats housed in Shelter B were given pelleted litter prior to data collection, whereas cats housed in Shelter A were given clay litter. Cats at both locations were given the same clumping clay cat litter during the study. Villeneuve-Beugnet & Beugnet [63] found that cats significantly preferred clay litter over pelleted litter. Additionally, an earlier study by Borchelt [64] found that when given the choice between finer clay litter and larger particle litter, cats rarely chose the larger particle litter. It is possible that the cats in Shelter B did not like the pelleted litter they were given prior to the study and held their pee when initially offered it. Thus, when they were given the preferred clay litter on the first day of the study, they peed more than expected.

To the authors’ knowledge, this is the first empirical study to utilize FAS Spectrum in its full form. A greater FAS Spectrum Score was significantly correlated with a greater CSS, suggesting it may be measuring a similar state in cats. A lower CSS was significantly related to more days since entering the study and experiencing a kennel change as well as coming into the shelter as a stray. This supports previous literature which has found that CSS decreases as days in the shelter increase [17,43] and that owner surrendered cats had higher CSS score as compared to cats who enter the shelter as a stray [38]. Interestingly, we did not find a relationship between CSS nor FAS Spectrum scores and urine output. CSS and FAS scores were collected in the morning during daily husbandry. Previous research found no relationship between morning CSS and urinary cortisol: creatine ratio (a physiological indicator of stress) [43]. In the present study, there was at least one researcher present in the room during the video recording for CSS and FAS Spectrum analysis. It is possible that the CSS and FAS Spectrum reflected the cats’ fear associated with the presence of the unfamiliar person and camera, and not their overall stress. Despite its widespread use, researchers have suggested that CSS may be an indicator of fear, not stress [44]. The cats were not exposed to the researcher recording the video prior to the first recording. Thus, the decrease in CSS and FAS scores across days in the study could also reflect an acclimation to a previously unfamiliar person. The lack of relationship between CSS and food and water intake and urine output seem to provide additional support for the idea that CSS is likely an indicator of fear, not stress.

One potential limitation of the current study is that CSS and FAS were only measured once daily using a relatively short video clip. It is possible that this brief observation period was not indicative of the cats’ overall stress (or fear). Previous methods of scoring CSS have included both live coding[10,17,38,41,42,47,65–69] and the use of videos [47,70–73]. The duration and frequency of the observations used to score CSS have also varied from brief one time observations [68] to observations every 30 minutes for seven hours [42]. Although many studies have averaged scores from multiple observations into one overall score [17,66,67,70–73], the studies do not report the intraindividual variability. Thus, it is difficult to determine whether it is necessary to have multiple observation periods, or if one is sufficient. If researchers continue to use observational measures such as CSS and FAS Spectrum, future research should explore the best practices surrounding the collection of these measures.

Although group did not predict litter clump weight, when looking specifically at cats who received petting, cats who made contact with the researcher’s hand and subsequently received petting had significantly greater urine output and lower CSS. Similarly, Gourkow and colleagues [13] found that cats who responded well to petting had a greater increase in secretory immunoglobulin A (SIgA) compared to cats who responded negatively. Previous research has found a wide range of human sociability in both shelter and pet cats [74]. The combination of previous findings and the findings in the present study seem to suggest that certain cats might benefit from human attention. However, it is also possible that cats who were less stressed were more likely to touch the researcher’s hand. Future research should explore the potential causal relationship between stress and social contact in cats. The beginning of the petting used in the current study (approach quietly and present hand nearby cat) resembles the Approach Test [75]. In the Approach Test, an individual approaches the cat in a slow fluid motion and presents their hand approximately 20 cm from the cat. Cats are labeled as “Contact Possible” if they do not retreat from the hand. Cats who retreat from the hand are labeled as “No Contact Possible.” Although their results were not statistically significant, Grant and Warrior [76] found that two weeks of brief clicker training caused four out of six cats who previously failed the approach test to pass the Approach Test. Future research could benefit from expanding the work on clicker training No Contact Possible cats to determine if their urine output increases with petting following the intervention.

## Conclusions

The results of the current study indicate that the addition of extra space and daily petting does not significantly impact urine output nor CSS in shelter cats. However, shelter location, defecating, and food and water consumption predict urine output and likely cat stress. Although receiving petting did not have an overall impact on urine output and CSS, cats who made contact with the researcher’s hand and solicited petting had higher urine output and lower CSS. Future research should explore not only the relationship between cat stress and social contact, but also shelter-specific factors related to urine output and cat stress.

## Supporting information

S1 Data Full dataset. (CSV)
